# The Association between Artificial Intelligence Awareness and Employee Depression: The Mediating Role of Emotional Exhaustion and the Moderating Role of Perceived Organizational Support

**DOI:** 10.3390/ijerph20065147

**Published:** 2023-03-15

**Authors:** Guanglu Xu, Ming Xue, Jidi Zhao

**Affiliations:** 1 School of Business, Nanjing University of Information Science & Technology, Nanjing 210044, China; 2 School of Business Administration, Shanghai Lixin University of Accounting and Finance, Shanghai 201620, China; 3 School of Public Administration, College of Economics and Management, East China Normal University, Shanghai 200062, China

**Keywords:** artificial intelligence awareness, emotional exhaustion, depression, perceived organizational support

## Abstract

The combination of artificial intelligence (AI) technology with the real economy has dramatically improved the efficiency of enterprises. However, the replacement of AI for employment also significantly impacts employees’ cognition and psychological state. Based on the Conservation of Resources Theory, the relationship between AI awareness and employee depression is explored in this article while examining the mediating role of emotional exhaustion, as well as the moderating role of perceived organizational support. Based on a sample of 321 respondents, the empirical results show that (1) AI awareness is significantly positively correlated with depression; (2) emotional exhaustion plays a mediating role between AI awareness and depression; (3) perceived organizational support negatively moderates the relationship between emotional exhaustion and depression; (4) perceived organizational support negatively moderates the mediating role of emotional exhaustion between AI awareness and depression. The research conclusions provide a theoretical basis for organizations to take measures to intervene in the negative impact of changes in AI technology on employees’ mental health.

## 1. Introduction

Artificial intelligence (AI) technology has rapidly advanced in recent years and has been widely implemented in various sectors, allowing enormous value to be generated through businesses [1]. However, while the development of AI has given a strong impetus to economic growth and improved the efficiency of economic development, it has also significantly impacted the labor market [2]. Scholars predicted that AI would replace 47% of jobs in the United States in the coming decades [3]. Nearly 55% of front-line manufacturing jobs in China are easily replaceable [4]. In reality, manufacturing enterprises have begun to replace labor with AI machines. As early as 2016, the Foxconn Kunshan Factory replaced 60,000 workers with a batch of AI machines [5]. As some positions will be replaced, AI technology will have a significant impact on employees’ career development, making it an inescapable stressor in contemporary workplaces [6]. In response to this problem, some scholars have called for attention to be paid towards employees’ cognition and coping behavior with AI technology [7]. Brougham and Haar put forward AI awareness to describe the extent to which an employee views the likelihood of AI technology impacting their future career prospects [7]. After this concept was put forward, many scholars studied the influence of AI awareness on employees’ psychological state and behavior. It mainly includes four aspects. First, AI awareness has a positive impact on employees’ psychological state, such as improving employees’ internal work motivation [8]. Second, AI awareness has a negative impact on employees’ psychological state, such as enhancing employees’ job insecurity [9,10], causing employee job burnout [11] and depression [7], reducing the career competency of employees [11], and negatively affecting employees’ organizational identity and career satisfaction [7,11]. Third, AI awareness has a positive impact on employee behavior, such as improving employees’ innovative behavior [8,12,13], promoting employees’ career exploration behavior [14], positively affecting employees’ work engagement [15], and encouraging active learning and task crafting [13]. Fourth, AI awareness has a negative impact on employee behavior, for example, increasing employees’ cynicism and turnover intention [1,6] and promoting employees’ knowledge hiding [16].

Lazarus theorized that stressors can affect employees’ psychological state and behavior depending on their cognitive appraisal [17]. When stressors are appraised as challenges, individuals will take more positive measures to deal with them, promoting their well-being. However, if stressors are appraised as threats, individuals will take more negative measures to deal with them, harming their well-being [18]. As an important stressor, AI awareness reflects employees’ threat appraisal of AI technology, which may be an important factor affecting their mental health. From the current research, it can be seen that there are still few studies on the relationship between AI awareness and employee mental health. Only Brougham and Haar discussed the correlation between AI awareness and employee depression [7]. However, there is no research on the mediating and moderating mechanisms of AI awareness and employee depression. Depression is an important indicator of mental health, leading to social and occupational dysfunctions [19], bringing significant psychological pain to individuals while seriously endangering their interpersonal relationships, social functions, and quality of life [20]. At the same time, employee depression substantially threatens the normal functioning of an organization through low productivity, employee absenteeism, and poor morale [21]. As a result, the purpose of this article is to discuss the mediating and moderating mechanisms between AI awareness and employee depression. The research significance of this article lies in the following: firstly, it helps to reveal the ways and boundary conditions of the influence of AI awareness on employee depression; second, the research conclusion provides a theoretical foundation for organizations to develop policies to prevent employee depression that may arise during AI implementation.

According to Brougham and Haar, AI technology threatens individuals’ total career growth, making it difficult to achieve their career goals. Furthermore, the application of AI technology in an organization will force employees to face the risk of being replaced by AI devices and being undervalued and not regarded highly by their employer, therefore, lowering their organizational status [7]. According to the Conservation of Resources (COR) Theory, people actively acquire, maintain, or protect valuable resources recognized by them. Career development and organizational status are important resources for individuals [22]. When employees realize that AI technology threatens their career development or organizational status, these resources face a considerable risk of depletion, which often leads to the emotional exhaustion of individuals [23], thus increasing the risk of depression in employees [24,25,26]. Therefore, emotional exhaustion may be a key mediating mechanism between AI awareness and employee depression.

AI awareness is positively related to individual emotional exhaustion, which is viewed as the process of the wearing out and wearing down of a person’s energetic resources [27] and is positively related to depression. However, perceived organizational support, as a psychological resource, would give employees a positive emotional experience [28], which can compensate for the loss of resources in daily work and alleviate the negative impact of the excessive consumption of resources [29,30]. Therefore, perceived organizational support may reduce the correlation between emotional exhaustion and employee depression. Furthermore, perceived organizational support may reduce the indirect correlation between AI awareness and employee depression via emotional exhaustion.

**Figure 1:**
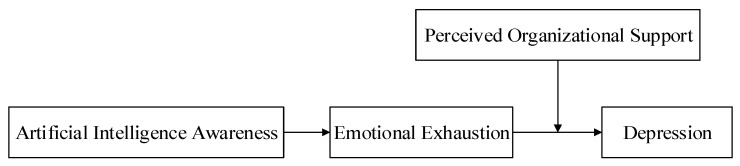
The theoretical framework.

Based on the above analysis, a moderated mediation model will be built in this article to analyze the relationship between AI awareness and employee depression, including the mediating effect of emotional exhaustion between AI awareness and employee depression. In addition, we also examined the moderating effect of perceived organizational support on the relationship between emotional exhaustion and employee depression, as well as the indirect relationship between AI awareness and employee depression via emotional exhaustion. The theoretical framework of this article is shown in Figure 1. This research is helpful for deeply understanding the influencing ways and boundary conditions of AI awareness on employee depression, helping enterprises formulate related policies to prevent employee depression and promote AI reform smoothly.

## 2. Theory Background and Hypotheses

### 2.1. AI Awareness and Depression

The main features of depression include sadness, guilt or low self-worth, fatigue and poor concentration, loss of pleasure or interests, and poor sleep or appetite [31]. Regarding the risk factors of depression, several researchers have found that losing social and material resources is positively associated with depression [32,33,34].

According to the COR Theory, there are four kinds of resources: object resources (such as houses and cars), condition resources (such as marriage, status, and employment), personal resources (such as critical skills and personal traits), and energy resources (such as credit, knowledge, and money) [27].

The introduction of AI technology will threaten individual resources, mainly regarding the following aspects. First of all, it may replace some occupations, making it difficult for individuals to achieve their career goals [7], reducing their conditional resources. Secondly, the wage gap for routine/unconventional tasks has widened within organizations [35], leading to a relative decrease in some individuals’ income, which refers to energy resources. Thirdly, the use of a large number of AI machines will change the required skill structure of workers. Workers’ existing skill set is no longer fit for new job requirements, so they need to invest time and energy to learn new skills, which also means a loss of original personal resources. Fourthly, after the application of AI, it may lead to the inconsistency between employees’ knowledge and skills needed for new tasks and their own knowledge and skills, which will have a negative impact on employee’s self-concept–job fit and reduce individuals’ conceptions of the self, such as self-esteem [36]. Self-esteem belongs to personal resources [29]. Therefore, the application of AI will lead to the loss of personal resources. Fifthly, the application of AI makes some workers’ positions in enterprises precarious, which may lead to a perception that employers ignore them and lower their organizational status [7], meaning a loss of condition resources. Sixthly, the replacement of positions by AI applications will cause employees to worry about losing their jobs, generating job-stress-related presenteeism, which siphons off cognitive energy that could otherwise be used to focus on their work [37,38]. In this case, employees’ energy resources also face the possibility of loss.

The COR Theory holds that the loss of resources will put great pressure on individuals [29]. Stress was a risk factor for depression [39]. Hobfoll et al. posited that resource loss is an important cause of various negative consequences, including depression [40]. Brown and Andrews reported that about 90% of the depression cases they studied were related to losses, except for those that might be a personality disorder [22].

Therefore, the greater the threat that AI technology poses to an individual’s career development, the stronger their AI awareness is, the greater the risk of resource loss is, and the higher their risk of depression will be.

Based on the above analysis, we put forward hypothesis 1: AI awareness will be positively associated with depression.

### 2.2. The Mediating Role of Emotional Exhaustion

Emotional exhaustion refers to emotional overwork and exhaustion caused by work, which is manifested as physical fatigue as well as psychological and emotional drain [41]. COR Theory holds that individuals tend to acquire, maintain, and preserve resources, who will be under pressure when faced with the threat of resource loss, actual loss, and failure posed to their acquisition of resources after investing in them [42,43]. Based on the above analysis, the introduction of AI threatens individual career development and makes individuals face a considerable risk of resource loss, becoming an important stressor. As a stressor, the influence of AI on the individual psychological state and behavior depends on their cognitive evaluation. When some individuals evaluate AI technology as a challenge, they are more willing to have a positive response, and if they evaluate AI technology as a threat, they will have a negative response [44]. AI awareness means that individuals think that AI technology threatens their career development. Therefore, the stronger the AI awareness is, the greater the possibility of a negative response will be [7]. When adopting a negative coping style, individuals will want to avoid AI technology, thus reducing their motivation [45]. In this case, the gap between individual work skills and knowledge and the requirements of the new posts after AI changes will become larger, so people’s jobs will be more threatened. As a conditional resource, a job provides compensation and a sense of self-esteem [43]. If the existence of jobs is threatened, the resources can only be made to face the risk of further losses. When individuals think that they are facing a loss of resources and cannot replenish them in time, it will lead to emotional exhaustion [22,46]. Additionally, after the introduction of AI, when realizing that their jobs are threatened, individuals usually have fear and anxiety about their future [47]. These negative emotions further reduce an individual’s ability to save or obtain resources to cope with this situation, leading to more stress, strain, and emotional exhaustion [48]. Therefore, we put forward hypothesis 2: AI awareness will be positively associated with employees’ emotional exhaustion.

Emotional exhaustion is conceptualized as representing a deficit in resources, and resource deficits lead to individual psychological problems [49,50]. According to COR Theory, individuals can improve their well-being by preserving and acquiring essential resources. If the key resources are lost, individuals will have destructive emotions, such as tension, stress, and anxiety [50], which are important risk factors for depression [51]. Many studies have shown that emotional exhaustion is highly correlated with depression. It is also found in studies on Chinese students that emotional exhaustion is positively related to depression [52,53]. Depressive disorders are also found to be positively predicted among teachers in primary and secondary schools as well as nurses with emotional exhaustion [54,55]. Therefore, we put forward hypothesis 3: emotional exhaustion will be positively associated with employee depression. Combining Hypothesis 2 with Hypothesis 3, we can put forward Hypothesis 4: emotional exhaustion will mediate the relationship between AI awareness and depression.

### 2.3. The Moderating Role of Perceived Organizational Support

Perceived organizational support refers to employees’ general belief that their organizations value their contributions and care for their well-being based on their perception of how organizations reward their work performance and meet their social as well as emotional needs [56]. Perceived organizational support can be regarded as a psychological resource leading to positive emotional experiences because employees feel the support, understanding, and affirmation of their abilities from their colleagues as well as leaders [28]. Emotional exhaustion was viewed as the process of the wearing out and wearing down of a person’s energetic resource [27]. According to COR Theory, psychological resources can compensate for the loss of resources in daily work and alleviate the negative impact of over-consumption [29,30]. Therefore, perceived organizational support can reduce the influence of emotional exhaustion on depression. Studies have also shown that people with higher perceived organizational support experience fewer physical and psychological problems, such as depression and anxiety [57,58].

The buffering model of social support also holds that a high degree of social support can buffer the impact of stress on depression [59], while low social support will increase individual susceptibility to depression [60]. Emotional exhaustion is a typical stress response [61]. Therefore, social support can reduce the negative impact of emotional exhaustion on depression [62,63]. The mechanism may be that social support can improve individual self-esteem, thus buffering the effect of emotional exhaustion on individual depression [53,64].

According to the above analysis, it can be speculated that organizational support, as a social support from organizations, can alleviate the depression caused by emotional exhaustion. The higher the perceived organizational support is, the lower the impact of emotional exhaustion will be on depression. Therefore, we put forward Hypothesis 5: perceived organizational support will negatively moderate the associations between emotional exhaustion and depression.

Based on Hypothesis 4 and Hypothesis 5, it can be speculated that the higher the perceived organizational support is, the lower the mediating relationship between AI awareness and depression will be through emotional exhaustion. Therefore, Hypothesis 6 is that perceived organizational support will negatively moderate the mediating role of emotional exhaustion between AI awareness and depression.

## 3. Methods

### 3.1. Procedure and Sample

The research data were collected from “Credamo” (https://www.credamo.com/, accessed on 25 December 2022). In order to avoid the influence of common method biases, a two-stage survey with an interval of about two weeks was adopted. The demographic variables, AI awareness, and perceived organizational support of samples were investigated in the first stage. We distributed 692 questionnaires and collected 447 after invalid respondents (identified by trap questions and reverse questions) were eliminated. In the second stage, questionnaires were distributed to the 447 respondents in the first stage, mainly to measure their emotional exhaustion and depression. After eliminating invalid questionnaires, 321 respondents were collected and matched. In terms of the features of respondents, men accounted for 47% and women accounted for 53%. The ages of the respondents were mainly distributed between 20 and 58 years old, and the distribution between 25 and 40 years old was concentrated, accounting for 89.1%. Respondents with a Bachelor’s degree accounted for 80.4%, undergraduates and above accounted for 11.2%, and the others accounted for 8.4%. Respondents came from a variety of occupations, including finance/auditing, management, technology/R&D, human resource management, production workers, clerical/office staff, administration/logistics staff, salespersons, customer service, professionals (such as accountants, lawyers, architects, healthcare workers, journalists), PR people, teachers, etc. With the popularization of AI, these occupational groups are more or less affected by AI applications, thus ensuring the effectiveness of sample selection.

### 3.2. Measures

The relevant variable measurement scales used in this study are from mature scales used in internationally renowned journals. We translated these scales into Chinese through a back-translation procedure proposed by Brislin [65] and fine-tuned some items according to Chinese daily expression habits. The key variables were scored on a 7-point Likert scale.

AI Awareness: The scale developed by Brougham and Haar [7] was adopted, and the original scale was appropriately revised according to the background of AI studied in this article. Specifically, “smart technology, automation, robotics and AI” in the original scale were briefly described as AI. There were four items on the scale, one of which was: “I am personally worried about my future in my organization as AI is replacing employees” (see Appendix A). In this study, Cronbach’s α = 0.91.

Emotional Exhaustion: The scale developed by Watkins et al. [66] was used. There were three items on the scale, one of which was: “I feel burned out from my work” (see Appendix A). In this study, Cronbach’s α = 0.91.

Perceived Organizational Support: The scale adapted by Shanock and Eisenberger [67] was used. There were six items on the scale, one of which was: “My work organization values my contributions to its well-being” (see Appendix A). In this study, Cronbach’s α = 0.82.

Depression: The scale developed by Dhir et al. [68] was adopted. There were five items on the scale, one of which was: “I have felt lonely” (see Appendix A). In this study, Cronbach’s α = 0.87.

Control Variables: To avoid other variables interfering with the relationship among the core variables in this article, we took individual personality variables as control variables, such as gender, age, education level, and occupation. According to the characteristics of occupations, we divided the sample occupations into physical occupations (production workers), administrative service occupations (administration/logistics staff, clerical/office staff), marketing occupations (salespersons, PR people, customer service), professional occupations (human resources management, finance/auditing, teachers), professionals (such as accountants, lawyers, architects, healthcare workers, journalists), technology research and development occupations (technology/R&D personnel), and management occupations (management). Because the occupation is a classified variable, that is, a nominal variable, its own coding has no practical quantitative relationship, and it only represents the differences between categories. According to Xie [69], in regression analysis, nominal variables cannot be directly included in the regression model as independent variables, and they must be transformed into a set of corresponding virtual variables. Therefore, this article takes the physical occupation as the reference class and uses the other five kinds of virtual variables in the research model. Studies have shown that AI application would affect employees’ work attitude and behavior. There are differences while applying AI in different enterprises, and there are bound to be differences in employees’ work attitude and behavior. Therefore, AI application was taken as a control variable in this article and the measurement of Wang et al. [70] was adopted. There were four items, one of which was: “Compared with manpower, the application range of AI in my unit will be wider and wider.” Cronbach’s α = 0.75.

## 4. Results

### 4.1. Confirmatory Factor Analysis

Confirmatory factor analysis was used to test the validity of discrimination among four variables: AI awareness, emotional exhaustion, depression, and perceived organizational support. As shown in Table 1, the data analysis results show that the four-factor model has the best fit with the samples (CFI = 0.94, TLI = 0.93, RMSEA = 0.07, SRMR = 0.04), compared with which other models have a poor fit and have passed the chi-square test with a significance level of 0.001, indicating that the measurement in this study has good discrimination validity.

### 4.2. Common Method Bias

To avoid the influence of common method bias on the research conclusion, Harman’s single factor test was used to test the common method bias. The results showed that the proportion of the total variance in the first factor accounted for 36.75%, which did not exceed the threshold of 40%, indicating that there was no serious common method bias in the data [71].

### 4.3. Descriptive Statistics and Correlation Analysis

The mean, standard deviations, and correlations among the research variables are shown in Table 2. The results show that there is a significant positive correlation between AI awareness and depression (r = 0.17, *p* < 0.01), between AI awareness and emotional exhaustion (r = 0.31, *p* < 0.01), and between emotional exhaustion and depression (r = 0.52, *p* < 0.01).

### 4.4. Hypotheses Testing

Firstly, multiple linear regression was used in this article to test Hypothesis 1, Hypothesis 2, Hypothesis 3, and Hypothesis 4. In the process of regression, the gender, age, education level, AI application, and occupation were taken as control variables. The specific regression results can be seen in Table 3.

It can be seen from Model 3 that AI awareness was positively associated with employee depression (β = 0.18, *p* < 0.01); thus, Hypothesis 1 was supported. It can be seen from Model 1 that AI awareness was positively associated with employees’ emotional exhaustion (β = 0.34, *p* < 0.001); thus, Hypothesis 2 was supported. It can be seen from Model 2 that emotional exhaustion was positively associated with employee depression (β = 0.52, *p* < 0.001); thus, Hypothesis 3 was supported. It can be seen from Model 4 that emotional exhaustion was positively associated with employee depression (β = 0.51, *p* < 0.001), but AI awareness was not significantly associated with employee depression again. According to the method proposed by Baron and Kenny [72], it could be judged that emotional exhaustion completely mediated the relationship between AI awareness and employee depression. Thus, Hypothesis 4 was supported.

To ensure the robustness of the research results, referring to Hayes’ method [73], we used SPSS PROCESS macro 3.4 to verify the mediating role of emotional exhaustion. In the process, gender, age, education level, occupation, and AI application were taken as control variables. The results showed that the indirect effect of AI awareness on depression via emotional exhaustion was 0.11 and 95%CI = (0.06, 0.17), which did not include 0. Hypothesis 4 was again supported.

Then, according to the general test method, core variables were standardized by converting the raw scores into Z scores, including emotional exhaustion, perceived organizational support, and depression. The regression method was then used to test the moderating role of perceived organizational support, the results of which are shown by Model 5. Emotional exhaustion was positively associated with depression (β = 0.44, *p* < 0.001), while perceived organizational support was negatively associated with it (β = −0.18, *p* < 0.05). Then, the interaction term of emotional exhaustion and perceived organizational support was added to the independent variables of Model 5, and depression was regressed. The results showed that the interaction term of emotional exhaustion and perceived organizational support was significantly associated with depression (β = −0.22, *p* < 0.001), indicating that perceived organizational support moderated the relationship between emotional exhaustion and depression. Hypothesis 5 was supported.

**Figure 2:**
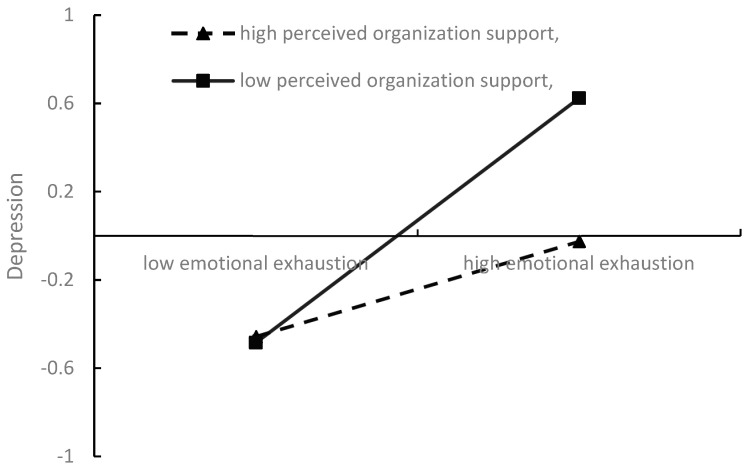
The moderating effect of perceived organizational support.

To further clarify the direction and size of the moderating effect, emotional exhaustion, perceived organizational support, and depression were standardized by converting the raw scores into Z scores in this study. The results of the simple slope test showed that when perceived organizational support was smaller, the relationship between emotional exhaustion and depression was positive and significant, and the effect was greater (β = 0.56, *p* < 0.001); when perceived organizational support was greater, the relationship between emotional exhaustion and depression was positive and significant, but the effect was smaller (β = 0.22, *p* < 0.01). The specific visualization results are shown in Figure 2, which shows that the higher the degree of perceived organizational support is, the lower the relationship between emotional exhaustion and depression will be, and perceived organizational support negatively moderates the relationship between emotional exhaustion and depression. Hypothesis 5 was supported again.

Then, we used SPSS PROCESS macro 3.4 to test Hypothesis 6, referring to Hayes’ method [73]. In the process, gender, age, education level, AI application, and occupation were taken as control variables. The results are shown in Table 4. When the perceived organizational support was smaller (mean standard deviation), the indirect effect between AI awareness and depression via emotional exhaustion was 0.12, 95%CI = (0.07, 0.19), and the confidence interval did not contain 0, indicating that the indirect effect was significant. When perceived organizational support was greater (mean + standard deviation), the indirect effect between AI awareness and depression via emotional exhaustion was 0.05, 95%CI = (−0.02, 0.12), and the confidence interval contained 0, indicating that the indirect effect was not significant. When the values of perceived organizational support were different, the difference in indirect effect was −0.07, 95%CI = (−0.16, −0.01), and the confidence interval did not contain 0, which indicated that there were significant differences in the indirect effect. The index of moderated mediation = −0.06, 95%CI = (−0.12, −0.01). These results showed that there was a significant moderated mediating effect, and the higher the degree of perceived organization support, the lower the indirect effect between AI awareness and depression via emotional exhaustion. Hypothesis 6 was supported.

## 5. Discussion

The impact of AI technology on employees was mainly discussed from two aspects in previous studies. First of all, from the perspective of technological application itself, studies examined the impact of AI technology application on employment [3], negative emotions [74], and job insecurity [70]. Secondly, starting from employees’ cognition of threats posed by technological application, studies examined the effect of AI awareness on job insecurity [6], job satisfaction [7], and turnover intention [1]. However, the mediating and moderating mechanism of AI awareness on employee depression was systematically rarely investigated. Based on COR Theory, the relationship between AI awareness and employee depression was discussed in this article while examining the mediating role of emotional exhaustion and the moderating role of perceived organizational support.

Based on a sample of 321 respondents, the empirical results showed that: first, AI awareness was positively associated with employee depression. This conclusion was consistent with that of previous research [7], and it was verified that psychological disorders (such as depression) could also be triggered among employees who perceived AI technology to threaten their career development in a Chinese sample. In addition, it was found in this study that the control variable, AI application, was negatively associated with employee depression, which was inconsistent with the results of previous studies [74]. A reason behind this phenomenon may be that the relationship between AI application and depression is a result of controlling the influence of AI awareness. Therefore, the relationship between AI application and employee depression here is more of a positive relationship formed by the opportunities brought by AI technology to employees’ career development. Second, emotional exhaustion plays a mediating role between AI awareness and employee depression, showing that the threat of AI technology to employees’ mental health mainly comes from the threat of AI changes to employee resources, which is consistent with the results of previous studies, showing that emotional exhaustion plays a mediating role in the process of stress factors and leads to employee depression [53,55]. In addition, when studying the impact of AI awareness on employees’ psychological state and work attitudes, Brougham and Haar posited that AI awareness was related to a series of negative consequences [7]. However, they did not confirm this mechanism based on data, and the conclusions of this article indirectly supported their inferences. Third, perceived organizational support negatively moderated the relationship between emotional exhaustion and depression. Furthermore, perceived organizational support negatively moderated the mediating effect of emotional exhaustion on the relationship between AI awareness and depression. This conclusion confirmed the view of COR Theory [29,30] that organizational support, as a resource, could supplement the resource loss of employees and reduce the negative impact of resource loss on employees’ stress reactions. In addition, as a form of social support, organizational support could function as a stress buffer and reduce the impact of stressful events on depression. The conclusion of this article also confirms the Stress Buffer Theory of social support [1].

### 5.1. Theoretical Implications

A moderated mediation model based on COR Theory is constructed in this research to explore the relationship between AI awareness and depression. The research conclusion has important theoretical significance for understanding the relationship mechanism between employees’ stress cognition and emotional response with the changes in AI technology.

First of all, previous scholars have discussed the relationship between AI awareness and employee depression based on a career-planning model [7]. However, they did not discuss the mediating mechanism between AI awareness and employee depression. In this article, the mediating mechanism of relation construction between AI awareness and employee depression is discussed from the perspective of COR Theory. In doing so, we expanded the interpretive perspective on the relationship between AI awareness and employee depression and an increased understanding of the mediating mechanisms between them.

Secondly, previous research lacked an examination of the buffering effect of organizational support in the relationship between AI awareness and employee depression, and only some research discussed the direct effect of organizational support on depression [30]. This research confirms the positive role of perceived organizational support in the process of employee stress relief and expands the cognition of boundary conditions between AI awareness and employee depression in the context of AI technological application.

Thirdly, the influence of AI awareness on employee depression was explored in a previous study [7]. However, the mechanism behind the relationship between AI awareness and employee depression has not been systematically investigated. A systematic model is put forward in this article to analyze the mediating and moderating mechanism between AI awareness and employee depression. Through analysis, the conclusion can help to deeply understand the impact and boundary condition between AI awareness and employee depression, which provide a theoretical basis for organizations to take measures to intervene in employee depression with changes in AI.

### 5.2. Management Implications

The integration of AI in industry can provide a market space for AI and improve the efficiency of the economy. However, it must be considered that the replacement of employment by AI technology will have a series of adverse effects on employees’ cognition and psychological state, which may become an important obstacle to the smooth implementation of this integration process. Therefore, measures must be taken to guide employees to correctly recognize the technological changes in AI, ease psychological problems arising from the process, ensure the support of employees for the changes in AI, and create a powerful internal environment for organizations to implement AI. The theoretical research results of this article provide a theoretical basis for policy making in organizations.

First of all, the research holds that AI awareness will increase employee depression. This conclusion shows that employees’ cognition significantly influences their psychological state. When employees perceive that AI is more threatening to their career development, their degree of depression is higher. Therefore, it is necessary to strengthen and guide employees to correctly recognize the influence of AI technology on career development through publicity. At the same time, companies should encourage employees to actively pay attention to the opportunities brought by AI technology to employees’ career development. Existing research also shows that with the introduction of AI technology, new employment opportunities will be created [75] while improving the job quality of employees [76]. Encouraging employees to actively recognize the opportunities for career development based on AI technology can make them actively respond to the impact of AI technology, thereby improving their career satisfaction and happiness as well as reducing their depression.

Secondly, the research shows that emotional exhaustion is an important mediating mechanism between AI awareness and employee depression. According to COR Theory, in the face of AI technological changes, although opportunities and threats coexist, due to the uncertainty of opportunities, employees tend to conserve resources to respond to threats rather than investing in them to respond to opportunities [33]. In this case, it is impossible for employees to keep up with the pace of AI technology, who are gradually eliminated with AI changes, encountering a gradual loss in resources and making them fall into a loss spiral. Therefore, to avoid this scenario, companies must develop policies that guide employees to actively invest resources in response to AI changes, for example, changing knowledge and skill structures through learning. In addition, employees should improve their own self-efficacy regarding adapting to AI and learning the necessary new skills, so as to help them better master the knowledge and skills to adapt to AI to improve their job security, income, attendance, and organizational status, so as to reduce losses in resources and prevent emotional exhaustion.

Thirdly, the study has shown that perceived organizational support can effectively alleviate the indirect effect between AI awareness and depression via emotional exhaustion. This conclusion shows that although the threats posed by AI to employees’ career development will lead to emotional exhaustion, perceived organizational support, as an important psychological resource, helps employees recover from resource depletion. Therefore, organizations should strengthen their support for employees, recognize the efforts made by them in the process of AI transformation, and give them positive feedback. Meanwhile, organizations should formulate policies to help employees adapt to AI technological changes and enable them to strengthen their self-efficacy to such changes.

### 5.3. Limitations and Future Research Directions

Although a theoretical framework is proposed to preliminarily explore the relationship mechanism of AI awareness and employee depression in this study, there are still several deficiencies that need to be further improved in the follow-up research.

First of all, although a two-stage research method was used in this study to avoid common method bias, the core variables of emotional exhaustion and depression were still investigated simultaneously, making it possible that common method biases interfered with the research. In order to make the research results more objective, the use of the three-stage research method can be considered in follow-up research in the future, in which AI awareness, emotional exhaustion, and depression are evaluated by employees at three timepoints respectively.

Secondly, in the aspect of sample information collection, we collected the occupational information of the sample, ignoring the information of the industry where the sample is located. In future research on the relationship between AI awareness and depression, we can consider collecting information on the industry in which the samples are located, and also including the industry as a control variable in the research model to discuss whether the industry will affect the research conclusion. In addition, the existing research on the influence of AI awareness on employees’ psychological state and behavior often locks the research object in a specific industry, such as the service industry [11]. Future research can also focus on special industries when discussing the relationship between AI awareness and depression.

Thirdly, the relationship between AI awareness and employee depression is discussed in this article. AI awareness refers to how employees perceive that AI technology will threaten their career development [7]. Lazarus and Folkman believe that stressors impact employees’ psychological state and behavior depending on their cognition, including challenge and threat evaluations. Different cognitive evaluations on stress lead to different behavioral responses, which have different long-term effects on individuals [18]. Generally speaking, when evaluating stress as a threat, individuals are more likely to take a negative behavioral response; when evaluating stress as a challenge, they are more likely to have a positive behavioral response. This article is focused on the relationship between individual threat evaluations on AI technology and depression, but that between individual challenge evaluations on AI technology and depression is not investigated. In fact, according to the Transactional Theory of Stress, when evaluating AI technology as a challenge, individuals will be more inclined to take active measures to obtain more resources, which can theoretically alleviate depression. Therefore, we can further explore the relationship between individual challenge evaluations on AI technology and depression in the future.

Fourthly, although the mediating role of emotional exhaustion in the relationship between AI awareness and depression is examined according to COR Theory in this article, there may be other mediating mechanisms. Job insecurity is predicted based on AI awareness [6], which is associated with depression [77]. Since the application of AI has a negative impact on employees’ self-concept–job fit [36], it may negatively affect depression through the meaning of work [78]. Therefore, job insecurity and threatened self-concept–job fit may be essential mediating mechanisms between AI awareness and depression. In the future, we can consider further exploring the mediating roles of job insecurity and threatened self-concept–job fit between AI awareness and depression.

Fifthly, only the moderating effect of perceived organizational support between AI awareness and depression is examined in this article, ignoring other factors that can buffer the relationship between AI awareness and depression. Perceived organizational support, as a psychological resource from the outside world, can help individuals cope with stressful situations. However, in addition to the external resource support, internal resources can also play this role, such as individual self-efficacy and self-esteem, which can enable individuals to utilize a more active method to deal with the situation of emotional exhaustion brought by AI awareness, thus obtaining the supplement of resources while alleviating the negative impact of emotional exhaustion. In the future, we can further explore the moderating role of self-efficacy or self-esteem between AI awareness and employee depression.

## 6. Conclusions

According to COR Theory, the relationships between AI awareness and employee depression, the mediating role of emotional exhaustion, and the moderating role of perceived organizational support were explored in the current study. We found that AI awareness was positively associated with employee depression, emotional exhaustion played a mediating role in the relationship between AI awareness and depression, and employees’ perceived organizational support could alleviate the mediating role of emotional exhaustion between AI awareness and depression. Ultimately, we recommend that organizations take steps to mitigate the adverse effect of AI technology changes on employees’ mental health.

## Figures and Tables

**Table 1:** Confirmatory factor analysis results.

Model	Description	χ^2^	df	△χ^2^	△df	CFI	TLI	RMSEA	SRMR
1	Four-factor model	317.75	129			0.94	0.93	0.07	0.04
2	Three-factor model	1016.92	132	699.18 ***	3	0.73	0.69	0.15	0.14
3	Three-factor model	1079.32	132	761.58 ***	3	0.71	0.66	0.15	0.17
4	Three-factor model	704.60	132	386.86 ***	3	0.82	0.80	0.12	0.07
5	Two-factor model	1429.02	134	1111.27 ***	5	0.60	0.55	0.17	0.19
6	Two-factor model	1527.02	134	1209.28 ***	5	0.57	0.51	0.18	0.14
7	Single-factor model	1758.55	135	1440.80 ***	6	0.50	0.44	0.19	0.14

1. It is a hypothetical model. 2. AI awareness and emotional exhaustion are combined as one factor. 3. AI awareness and depression are combined as one factor. 4. Emotional exhaustion and depression are combined as one factor. 5. AI awareness and depression are combined as one factor; emotional exhaustion and perceived organizational support are combined as one factor. 6. AI awareness, emotional exhaustion, and depression are combined as one factor. 7. All variables are combined as one factor. △χ^2^ tests relative to Model 1. ***, represent *p* < 0.001.

**Table 2:** Descriptive statistics of variables and correlation matrix.

Variables	M	SD	1	2	3	4	5	6	7
1. Gender	0.47	0.50							
2. Age	30.54	5.37	0.10						
3. Education	5.02	0.58	0.08	0.02					
4. AI application	5.62	0.80	0.09	0.17 **	0.09				
5. AI awareness	3.03	1.36	−0.00	0.10	0.01	0.06			
6. Emotional exhaustion	2.87	1.37	−0.02	−0.12 *	−0.06	−0.29 **	0.31 **		
7. Perceived organizational support	5.76	0.65	0.12 *	0.07	0.14 *	0.28 **	−0.45 **	−0.49 **	
8. Depression	1.93	0.87	−0.02	−0.10	−0.00	−0.16 **	0.17 **	0.52 **	−0.40 **

* *p* < 0.05; ** *p* < 0.01; two-tailed tests. SD = standard deviation. Gender is coded 1 = male and 0 = female; Education level is coded 1 = primary school or below, 2 = junior high school, 3 = general high school/secondary school/technical school/vocational high school, 4 = college graduates, 5 = university graduates, 6 = Master, 7 = Ph.D.

**Table 3:** Multiple linear regression analysis results.

	Emotional Exhaustion	Depression				
	Model 1	Model 2	Model 3	Model 4	Model 5	Model 6
Gender	0.03	0.00	0.02	0.00	0.02	0.04
Age	−0.09 *	−0.05	−0.10	−0.05	−0.05	−0.05
Education level	−0.05	0.05	0.02	0.05	0.06	0.04
AI application	−0.28 ***	−0.01	−0.15 **	−0.01	0.02	−0.01
Occupations: Physical occupation						
Administrative service occupation	0.16 *	0.17*	0.26 **	0.17 *	0.15 *	0.16 *
Marketing occupation	0.00	0.01	0.01	0.01	0.01	0.02
Professional occupation	0.19 *	0.03	0.13	0.03	0.03	0.05
Technology research and development occupation	0.24 **	−0.02	0.11	−0.02	−0.01	0.02
Management occupation	0.06	0.08	0.11	0.08	0.08	0.09
AI awareness	0.34 ***		0.18 **	0.01		
Emotional exhaustion		0.52 ***		0.51 ***	0.44 ***	0.39 ***
Perceived organizational support					−0.18 **	−0.16 **
Emotional exhaustion×perceived organizational support						−0.22***
R2	0.25 ***	0.31 **	0.11 **	0.31 ***	0.33 ***	0.37 ***
ΔR2				0.20 ***		0.04 ***
F	10.11	13.74	3.78	12.46	13.75	15.17

* *p* < 0.05; ** *p* < 0.01; *** *p* < 0.001; two-tailed tests.

**Table 4:** Moderated mediating effect analysis results.

AI Awareness → Emotional Exhaustion → Depression				
Perceived Organizational Support	Effect	SE	95% Confidence Interval	
			LLCI	ULCI
Low perceived organizational support	0.12	0.03	0.07	0.19
High perceived organizational support	0.05	0.04	−0.02	0.12
Differences between high and low levels of perceived organizational support	−0.07	0.04	−0.16	−0.01

## Data Availability

The data presented in this study are available on request from the corresponding author.

## Appendix A

**Table A1:** Measurement items of key variables.

Variables	Items		Sources
Artificial Intelligence(AI) Awareness	1	I think my job could be replaced by AI	Brougham and Haar (2018) [7]
	2	I am personally worried that what I do now in my job will be able to be replaced by AI	
	3	I am personally worried about my future in my organisation due to AI replacing employees	
	4	I am personally worried about my future in my industry due to AI replacing employees	
Emotional Exhaustion	1	I feel emotionally drained from my work	Watkins et al. (2014) [66]
	2	I feel burned out from my work	
	3	I feel exhausted when I think about having to face another day on the job.	
Perceived Organizational Support	1	The organization values my contribution to its well-being	Shanock & Eisenberger(2006) [67]
	2	The organization strongly considers my goals and values	
	3	The organization really cares about my well-being	
	4	The organization is willing to help me when I need a special favor	
	5	The organization shows very little concern for me	
	6	The organization takes pride in my accomplishments at work	
Depression	1	I have felt lonely	Dhir et al. (2018) [68]
	2	I did not enjoy my life	
	3	I have felt myself unworthy	
	4	I have felt all the joy had disappeared from my life	
	5	I have felt my sadness was not relieved even with help of family/friends	
